# Repetitive Transcranial Magnetic Stimulation Improves Cognitive Impairment via the Regulation of White Matter Injury in Rats With Ischemic Stroke

**DOI:** 10.1002/brb3.71117

**Published:** 2025-12-07

**Authors:** Xiaoxia Hao, Qian Li, Can Luo, Xiangyu Tang, Haoyue Shao, Feng Guo

**Affiliations:** ^1^ Department of Rehabilitation Medicine, Tongji Hospital, Tongji Medical College Huazhong University of Science and Technology Wuhan China; ^2^ Department of Radiology, Tongji Hospital, Tongji Medical College Huazhong University of Science and Technology Wuhan China

**Keywords:** cognitive impairment, ischemic stroke, rTMS, SDF‐1α/CXCR4 axis, white matter injury

## Abstract

**Purpose:**

Poststroke cognitive impairment (PSCI) is a common functional disorder that occurs following stroke, but there are few effective therapies. Repetitive transcranial magnetic stimulation (rTMS) is a noninvasive neuromodulatory technique that has been used to improve cognitive function in stroke patients. Despite its widespread use in clinical research, the underlying mechanisms of rTMS are largely unknown. This study hypothesized that rTMS ameliorates PSCI by regulating white matter injury, which is of vital importance in cerebral ischemia.

**Method:**

An ischemic stroke rat model was created using transient middle cerebral artery occlusion. The extents of brain damage and white matter injury, including diffusion tensor imaging and diffusion tensor tractography, were evaluated using MRI. Behavioral tests, including the modified neurological severity score test and Morris water maze test, were also used. In addition, we preliminarily explored the potential role of SDF‐1α/CXCR4 by Western blot analysis and real‐time reverse transcription PCR.

**Finding:**

The results showed that 10 Hz rTMS promoted neurological recovery and cognitive deficits in ischemic rats. Additionally, 10 Hz rTMS alleviated cerebral infarct severity and attenuated white matter lesions. Furthermore, the expression levels of components of the SDF‐1α/CXCR4 axis influenced the effect of rTMS on ischemic stroke.

**Conclusion:**

This research provides further evidence that 10 Hz rTMS can alleviate white matter injury in affected brain regions and improve PSCI after ischemic stroke, potentially through the activation of the SDF‐1α/CXCR4 axis.

## Graphical abstract

10 Hz rTMS can alleviate white matter injury and improve functional impairments after ischemic stroke, which is potentially associated with the activation of the SDF‐1α/CXCR4 axis.

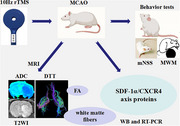

## 1 Introduction

Stroke is the second leading cause of mortality and the third leading cause of disability worldwide (Feigin and Owolabi 2023). High proportions of stroke survivors suffer from motor dysfunction and neuropsychiatric sequelae. Poststroke cognitive impairment (PSCI) is one of the major consequences of ischemic stroke and a major cause of long‐term disability. Approximately one‐third of stroke survivors have considerable cognitive impairment within a few months after stroke. Despite the relatively high prevalence of PSCI, the mechanism has not been fully elucidated, and few effective medical interventions are recommended. Exploring the neural mechanism of PSCI to optimize rehabilitation strategies is an urgent issue in basic and clinical research and has great practical theoretical significance and potential implications for clinical application.

Previous studies have focused mostly on focal or cortical infarction lesions, and few studies have explored the role of subcortical structural damage in the pathogenesis of PSCI. Recently, the disruption of white matter integrity was shown to cause cognitive decline (He et al. 2022). In addition to direct damage to brain structures caused locally by infarction, disruption of the integrity of white matter tracts affects structural connections between key brain regions, resulting in cognitive impairment (Schellhorn et al. 2021). Normal cognitive function is based on the structural integrity of extensive white matter tracts, and the occurrence of PSCI has been found to be strongly associated with the integrity of white matter fibers (Puy et al. 2018). As increasing evidence indicates that structural impairment after stroke is closely related to learning, memory, and cognitive functions (Schaapsmeerders et al. 2016; Zhu et al. 2022), the development of approaches aimed at promoting white matter recovery has become a promising research direction.

Repetitive transcranial magnetic stimulation (rTMS) is a noninvasive technique involving the repeated delivery of electric currents to a small area of the brain that is valuable technique for studying and treating focal cerebral ischemia. A growing number of studies have demonstrated its therapeutic effect on PSCI (Gao et al. 2023; Hong et al. 2021). One possible mechanism underlying the effects of rTMS that has been repeatedly reported is increased neural activity and plasticity. Moreover, achieving rehabilitation after stroke is partially dependent on the restoration of function mediated by proper structural organization in the brain (Hordacre et al. 2018). However, there are few data on the effects of rTMS on structural alterations, such as white matter damage, in PSCI. As elucidating the mechanism underlying the effect of rTMS on structural alterations is critical for developing better therapies for cerebral ischemia patients, the in‐depth neuroprotective effects of rTMS in PSCI require further exploration.

Stromal cell‐derived factor‐1α (SDF‐1α) and its affinity receptor C‐X‐C chemokine receptor type 4 (CXCR4) are highly expressed in the central nervous system and have beneficial effects on injured brains. SDF‐1α is found in all vertebrate tissues and plays important roles in cell proliferation, cell migration, and tissue‐specific physiological processes, including neuromodulation. In cerebral ischemia, SDF‐1α is secreted by astrocytes and endothelial cells around the infarct area, and CXCR4 is expressed on neural progenitors and stroke‐affected neuroblasts (Song et al. 2016). It has been shown to be significantly influenced by cerebral ischemia and mediates many factors relevant to adult neuroplasticity (Amanollahi et al. 2023; Han et al. 2019). Activation of the SDF‐1α/CXCR4 axis can reduce the infarct volume and improve memory functions effectively. In addition, SDF‐1α and CXCR4 have recently attracted significant attention because of their important role in supporting remyelination in white matter injury. However, research on the role of SDF‐1 and/or CXCR4 in remyelination has focused mainly on multiple sclerosis models (Beigi Boroujeni et al. 2020), and few studies have focused on cerebral ischemia and brain injury. Regardless, these studies may provide valuable insight into the function of SDF‐1α and CXCR4 in remyelination after white matter injury (Cheng et al. 2017). Hence, the SDF‐1α/CXCR4 axis is considered a key pathway and a promising target for achieving structural repair and neuroplasticity and thus alleviating PSCI after brain damage.

Our previous study revealed that 10 Hz rTMS can significantly increase the protein expression levels of SDF‐1α and CXCR4 in the striatum and promote the migration of neural stem cells (NSCs) in the subventricular zone (SVZ) after focal cerebral ischemia by regulating the SDF‐1α/CXCR4 axis (Deng et al. 2021). In addition, one study reported that electrical stimulation might modulate SDF‐1α concentrations in ischemic stroke (Morimoto et al. 2018). These insights into the effects of the SDF‐1α/CXCR4 axis on cerebral ischemia and plasticity reveal new evidence for the effectiveness of rTMS for the treatment of PSCI.

In this study, we explored the impact of rTMS on ischemic severity and white matter lesions by using magnetic resonance imaging (MRI). We investigated whether the SDF‐1α/CXCR4 axis plays a key role in rTMS‐mediated rescue of middle cerebral artery occlusion (MCAO)‐induced functional impairments. We hope to provide a theoretical basis for the use of rTMS to treat PSCI.

## 2 Materials and Methods

### 2.1 Animals and Experimental Groups

**FIGURE 1:**
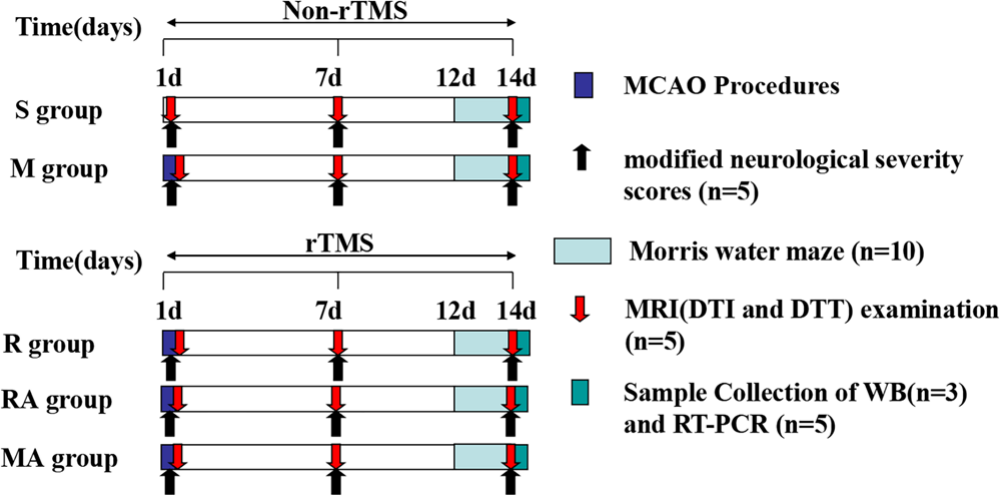
Experimental protocol.

Seven‐week‐old male Sprague‒Dawley (SD) rats (230260; Jingda Bioengineering Co., Ltd., Hunan, China) were used for this study. The rats were housed under a 12‐h light/dark cycle throughout the study and were provided ad libitum access to food and water (Huazhong University of Science and Technology Experimental Animal Center, Wuhan, China). Randomization was achieved by assigning a number to each rat, and a random number generator was used to divide the animals into experimental groups. The rats were divided into the sham (S), MCAO (M), rTMS+MCAO (R), rTMS+MCAO+AMD3100 (RA) and MCAO+AMD3100 (MA) groups. These five groups were subdivided into two subsets according to the time point: 7 and 14 days after MCAO. All groups except the S group underwent MCAO. The R group and RA group received rTMS treatment. The modified neurological severity scores (mNSSs) of five rats in each subgroup were assessed, and ten rats in each subgroup were subjected to the Morris water maze (MWM) test. Additionally, MRI was performed on the rats (n = 5) in the 7‐ and 14‐day subgroups. The protocol of this study is presented in **Figure** **1**.

### 2.2 MCAO

The rats were anesthetized with 5% isoflurane inhalation for induction and 2.5% isoflurane for maintenance. The right middle cerebral artery was occluded for 90 min, followed by reperfusion according to the method of Longa et al. (1989). The intraluminal suture occlusion method was performed as described in a previous study (Peng et al. 2019). Each rat was allowed to recover from anesthesia and was returned to its cage after the wound was sutured. During the surgical procedure, the rectal temperature was maintained at 37 ± 0.5°C with a heat lamp. In the S group, only the external carotid artery was ligated. The neurological deficit score (0, no deficit; 1, failure to stretch the left forepaw fully; 2, circling to the left; 3, falling to the left; and 4, no spontaneous walking or impaired consciousness) was assessed 4 h after the operation, and rats with a score of 2–3 were used as model animals (Longa et al. 1989).

### 2.3 rTMS Treatment

rTMS was delivered using a magnetic stimulator (YRD‐CCI, Wuhan Yiruide Medical Equipment New Technology Co., Ltd.) with a round animal coil (6 cm in diameter with 3.5 T peak magnetic welds). The coil was positioned perpendicular to the cortex approximately 5 mm to the right of the bregma, and stimulation was delivered to conscious rats every 24 h for 7 or 14 days after MCAO. The 10 Hz rTMS treatment protocol involved ten cycles of stimulation for 3 s followed by rest for 50 s (300 pulses per day). The stimulation intensity was set at 120% of the average resting motor threshold (RMT), namely, 26% of the maximum output of the stimulator. The treatment program was designed on the basis of our previous study according to its efficacy and feasibility (Guo et al. 2017).

### 2.4 Administration of AMD3100

The CXCR4 receptor antagonist AMD3100 (cat. no. HY‐10046; MedChem Express) was administered to the RA and MA groups. The rats in the RA and MA groups received AMD3100 (in saline; 2 mg/kg; i.p.) every other day beginning 2 days after MCAO until euthanasia, as described in previous reports (Deng et al. 2021).

### 2.5 MRI

MRI of the rats was performed at 1, 7, and 14 days after surgery by using a Discovery MR750 3.0 T MRI scanner (GE, USA) equipped with a radio frequency (RF) coil for animals. Each rat was lightly anesthetized and then placed in a prone position with its head in the middle of the coil. Multiple MRI sequences, including T2‐weighted imaging (T2WI) and diffusion tensor imaging (DTI) sequences, were acquired. The following sequence was used for T2WI: repetition time = 3.0 s, echo time = 85.0 ms, field of view (FOV) = 6 × 4.8 cm, slice thickness = 1.0 mm, and total scan time = 48 s. We visualized the lesion area on T2 weighted and diffusion‐weighted images, and manually depict the core of the infarcted area and the corresponding regions of the contralateral hemisphere on the diffusion‐weighted images using a GE postprocessing workstation. Then measure the apparent diffusion coefficient (ADC) values of the ischemic layer and the mirror contralateral region. The quantitative parameter obtained from the DWI is the ADC value. ADC value reflects the degree of water molecule diffusion within tissue, and lower ADC values typically indicate restricted diffusion due to cytotoxic edema in acute ischemic stroke. The severity of ischemia was determined by the ratio of ischemic area to contralateral mirror area (mean ± SD) (Yang et al. 2022).

DTI was performed using the following echo‐planar imaging sequence: diffusion direction = 20, repetition time = 2.0 s, NEX = 8, b value = 1000 s/mm^2^, number of slices = 14, slice thickness = 2.0 mm, field of view (FOV) = 4.1 × 4.1 cm, and total scan time = 5 min 38 s. We selected the lesion area within the same ROI. Diffusion tensor tractography (DTT) images were derived from original DTI images by the Diffusion Toolkit and TrackVis software. The fiber density and mean fiber length in the corresponding regions were obtained. The data are presented as the ratio of the ipsilateral value relative to the contralateral value.

### 2.6 mNSS

The neurological deficits of each rat were evaluated using the mNSS at 1, 7, and 14 days after the operation by an observer blinded to the experimental groups. The mNSS is a composite score of motor, sensory, reflex and balance functions. The neurological status was graded from 0 to 18, with 0 representing normal function and 18 representing the maximal deficit; higher scores indicate more severe neurological deficits (Chen et al. 2001; Salikhova et al. 2021).

### 2.7 MWM Test

On the 12th day after surgery, the learning and memory abilities of the rats were assessed using the MWM test. A circular water tank (150 cm in diameter and 50 cm deep) was filled with 23 ± 2°C water to a depth of 21 cm. A circular platform 10 cm in diameter and 20 cm in height was placed in the center of the target quadrant (quadrant III). The rats were subjected to two sessions of four place navigation trials per day at intervals of at least 4 h for 2 consecutive days (day 12 through day 13, 4 training trials per rat). On day 14, the platform was removed for the spatial probe trial. The latency to find the submerged platform, the number of platform crossings and the swimming paths were automatically recorded using a computer‐based image analyzer (MWM tracking system MT‐200, ChengDu Technology & Market Co., Ltd., Chengdu, Sichuan Province, China). The trends of latency time to find the submerged platform from the first time to the fifth time during the Morris water maze training trials are also shown in the dot plots. All the data were recorded by the same person, who was blinded to the grouping of the rats. The principles and technical details of the task were previously described (Han et al. 2013; Zhang et al. 2021).

### 2.8 Western Blot (WB) Analysis

Brain tissues from the ischemic hemisphere were microdissected from the 7‐ and 14‐day subgroups for WB analysis. Anti‐rabbit SDF‐1α (1:300; cat. no. bs‐4938R; Bioworld Technology, Inc.) and anti‐CXCR4 (1:300; cat. no. ab181020; Abcam) antibodies were used. Images were taken with an X‐ray film processor. Normalization was performed via a mouse monoclonal anti‐glyceraldehyde‐3‐phosphate dehydrogenase (GAPDH) antibody (1:500; Santa Cruz, Inc., CA, USA). The density of the bands was quantified using Gel‐Pro Analyzer version 4.0 software (Media Cybernetics, USA). The test was performed in triplicate.

### 2.9 RT‒PCR Analysis

Total RNA was isolated from microdissected ipsilateral brain tissue with TRIzol reagent (Invitrogen, Carlsbad, CA, USA). RT‒PCR was used to analyze the mRNA expression levels of SDF‐1α and CXCR4 in duplicate for each RNA sample. The RT‒PCR primer sequences are shown in Table 1. GAPDH was used as an internal control. The fold changes in SDF‐1α and CXCR4 expression were calculated relative to GAPDH expression using the comparative Ct method (2^−▲▲CT^).

**TABLE 1:** Primer information for RT‐PCR.

Name	Primer	Sequence	Size
Rat GAPDH	Forward	5‘‐ ACAGCAACAGGGTGGTGGAC‐3’	253 bp
	Reverse	5‘‐ TTTGAGGGTGCAGCGAACTT‐3’	
Rat Cxcr4	Forward	5‘‐ CGGTCATCCTTATCCTGGCT ‐3’	211 bp
	Reverse	5‘‐ CTCTTGAATTTGGCCCCGAG ‐3’	
Rat Sdf‐1	Forward	5‘‐ ATGCCCCTGCCGATTCTTTG ‐3’	114 bp
	Reverse	5‘‐ TTGTTGCTTTTCAGCCTTGC ‐3’	

### 2.10 Tissue Preparation

To avoid any potential impact on the results, we excluded rats that exhibited abnormal behaviors, such as seizures, during treatment. All the rats were immediately sacrificed, and brain tissues from the ipsilateral hemisphere, including the ischemic cortex and hippocampus, were microdissected for WB analysis and RT‒PCR.

### 2.11 Statistical Analysis

The data are presented as the means ± SEMs and were analyzed by SPSS 20.0 (IBM Corporation, Somers, NY, USA). GraphPad Prism version 6.0 software (GraphPad Software, Inc.) was used to generate graphs. Statistical comparisons of the mNSSs and MRI and RT‒PCR data were performed by two‐way ANOVA. Statistical comparisons of the MWM and WB data were performed by one‐way ANOVA. *p* < 0.05 was regarded as indicating statistical significance, and all numerical analyses were performed using GraphPad Prism software.

## 3 Results

### 3.1 rTMS Ameliorated Ischemic Damage After MCAO in Rats

**FIGURE 2:**
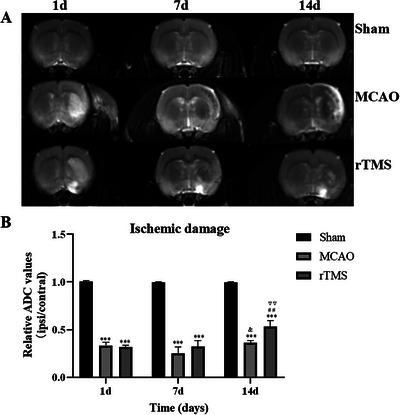
The effects of rTMS on ischemic damage in rats after surgery. (A) Representative T2WI maps of the results obtained at 1, 7, and 14 days postocclusion in all groups. (B) Comparisons of the relative degree of ischemic damage among the three groups at different time points and among the three time points within each group. ^***^ *p* < 0.001 vs. the corresponding S group; ^&^ *p* < 0.05 vs. the corresponding R group; ^##^ *p* < 0.01 vs. the 1‐day R group; ^▽▽^ *p* < 0.01 vs. the 7‐day R group.

The infarcted areas in the MCAO model rats presented high‐intensity signals on T2WI in the acute stage. Relative ischemic damage was evaluated through the ADC ratio values of the ischemic region to the corresponding region in the contralateral hemisphere (ipsi/contral) for all groups on the first day, seventh day and 14th day postocclusion. Representative maps of the three groups on days 1, 7, and 14 are shown in **Figure** **2A**.

No apparent lesions were detected in the sham group, the signals were similar between the cerebral hemispheres during the experiment (*p* > 0.05), and the relative ADC value was nearly 1. An analysis of variance revealed that the infarct severity was initially very similar between the M and R groups at 1 day postocclusion (M = 0.33 ± 0.07, R = 0.32 ± 0.05, *p* > 0.05). The infarct damage decreased, and the ratio of the ischemic region to the corresponding region increased over time in the rats. Although the difference in the ratios between the M and R groups was also not significant on day 7 (M: 0.25 ± 0.15, R: 0.32 ± 0.14, *p* > 0.05), on day 14, the ischemic tissues improved more in the R (0.55 ± 0.11) group than in the M (0.36 ± 0.05) group (*p* = 0.035) (**Figure** **2B**)

We also analyzed whether there were differences among the groups at different time points (**Figure** **2B**). The data revealed that ischemic damage was notably ameliorated in the R group on the 14th day compared with the R groups on the first (*p* = 0.0031) and seventh (*p* = 0.0042) days. In addition, the degree of ischemic damage increased 7 days after surgery. This might be because pseudo‐normalization generally occurs on the seventh day following cerebral ischemia. Pseudo‐normalization is typically observed around one week after ischemic stroke and most likely due to a combination of cytotoxic edema and the development of vasogenic edema (Kang et al. 2021). These results suggest that rTMS effectively alleviated ischemic severity in MCAO rats.

### 3.2 rTMS Increased the Expression Levels of Proteins in the SDF‐1α/CXCR4 Axis After Focal Cerebral Ischemia

**FIGURE 3:**
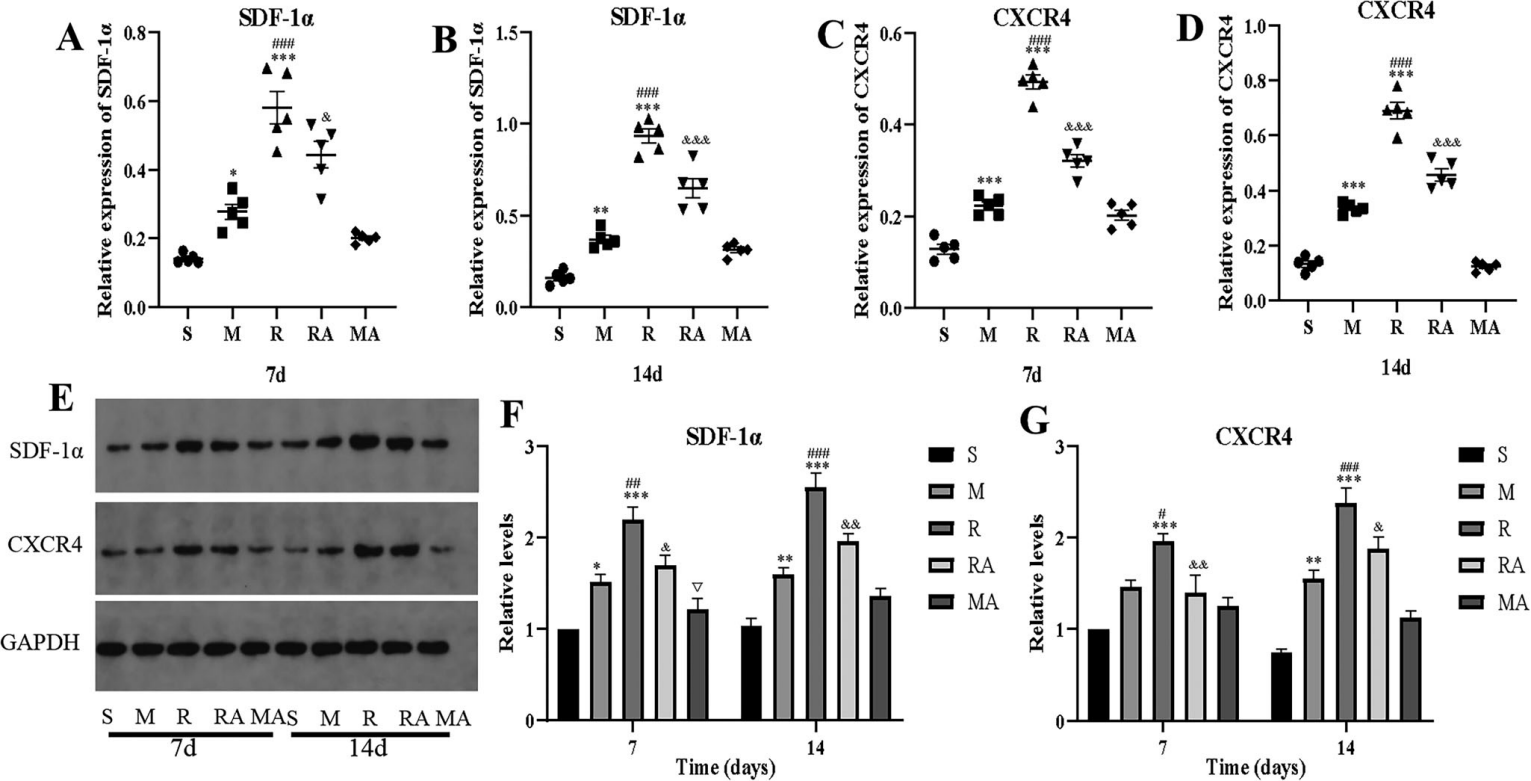
The effects of rTMS on the expression of SDF‐1α/CXCR4 axis components in ischemic tissues. (A–D) The ratio of the expression level of the target genes to that of GAPDH in all groups. (E) Gel electrophoresis of SDF‐1α and CXCR4. (F) RT‒PCR analysis of SDF‐1α mRNA levels. (G) RT‒PCR analysis of CXCR4 mRNA levels. ^*^ *p* < 0.05, ^**^ *p* < 0.01 and ^***^ *p* < 0.001 vs. the S group; ^#^ *p* < 0.05, ^##^ *p* < 0.01 and ^###^ *p* < 0.001 vs. the M group; ^&^ *p* < 0.05, ^&&^ *p* < 0.01 and ^&&&^ *p* < 0.001 vs. the R group.

To investigate the effect of rTMS on the SDF‐1α/CXCR4 pathway, WB analysis and RT‒PCR were performed to examine the expression of crucial signaling molecules in ischemic brain tissues at 7 and 14 days after MCAO. As shown in **Figure** 3A–C, the SDF‐1α and CXCR4 protein expression levels were markedly lower in the RA group than in the R group (*p* < 0.05) at both time points. Furthermore, SDF‐1α levels were significantly greater at both 7 and 14 days in the R group than in the M group (*p* < 0.001). Although the difference in CXCR4 expression between the M and R groups was not significant on day 7, CXCR4 expression was markedly greater in the R group than in the M group on day 14 (*p* < 0.001).

The same trend among the groups was also observed by RT‒PCR at both time points (all *p* < 0.05; **Figure** **3D** **–E**). These results suggested that the SDF‐1α/CXCR4 axis was inhibited by AMD3100 and that rTMS could upregulate the expression levels of SDF‑1α and CXCR4 in ischemic brain tissues.

### 3.3 rTMS Promoted White Matter Repair After Focal Cerebral Ischemia in Rats

**FIGURE 4:**
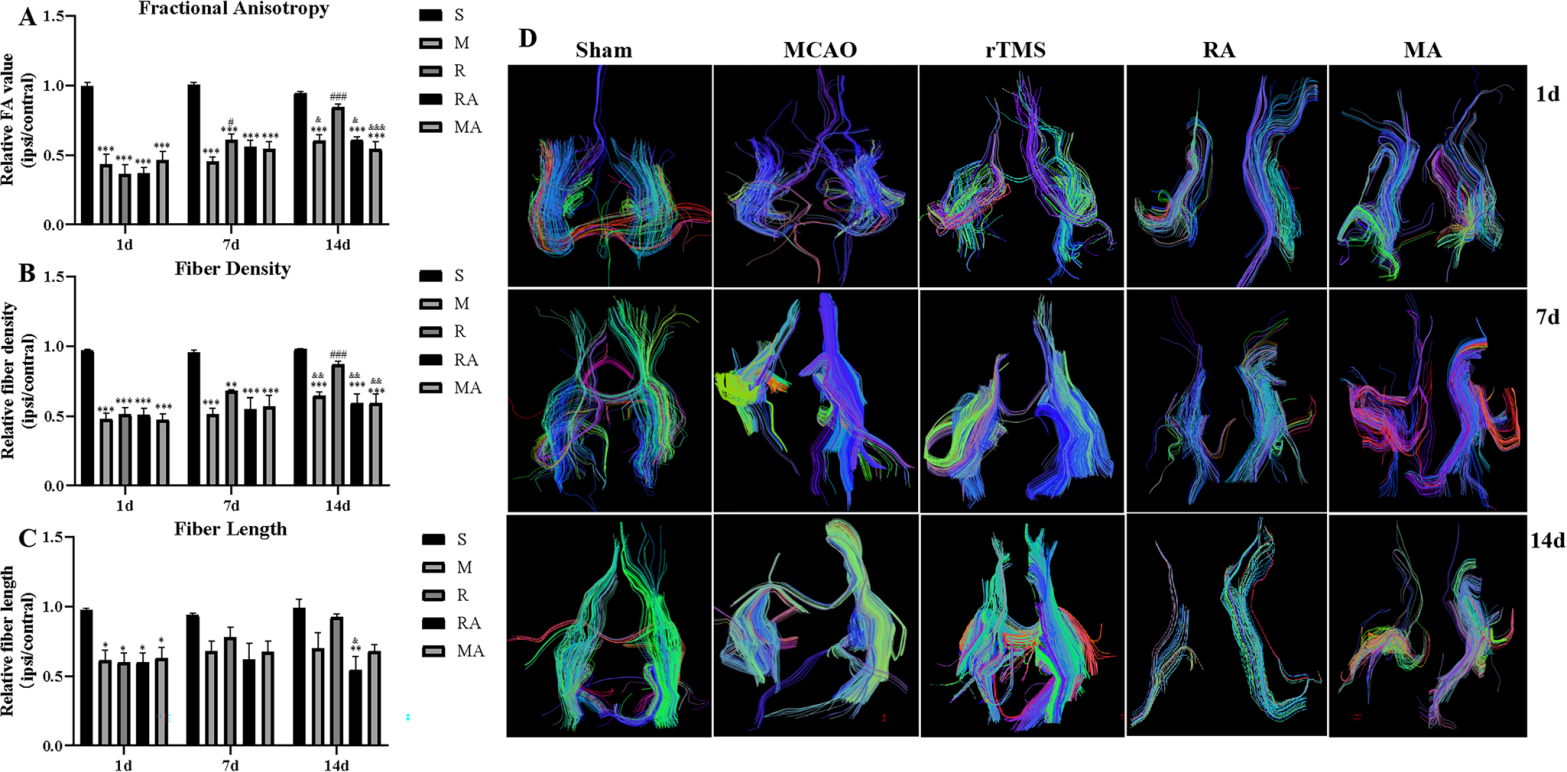
The effects of rTMS on white matter fibers in rats at 1, 7, and 14 days after surgery. (A) Comparisons of FA among the three groups. (B) Comparisons of the relative fiber density among the three groups. (C) Comparisons of the relative fiber length among the three groups. (D) Representative images of three‐dimensional fiber tracking at 1, 7, and 14 days postocclusion. ^**^ *p* < 0.01 and ^***^ *p* < 0.001 vs. the corresponding S group; ^&^ *p* < 0.05, ^&&^ *p* < 0.01, and ^&&&^ *p* < 0.001 vs. the corresponding R group; ^#^ *p*< 0.05, ^#^ *p* < 0.05, and ^###^ *p* < 0.001 vs. the 1‐day R group.

Fractional anisotropy (FA) represents the directionality of water diffusion within tissue, is a sensitive indicator of white matter injury (Yang et al. 2022), and is regarded as one of the most sensitive DTI parameters for assessing the integrity of white matter fibers after cerebral damage (Zhao et al. 2019). DTI revealed significantly reduced FA in the ipsilateral hemisphere in both the M and R groups compared with the S group at 1 day postocclusion, and there was no significant difference in the FA value between the M (0.44 ± 0.16) and R (0.36 ± 0.14) groups (*p* > 0.05). The FA value began to increase after the operation, and the FA value in the M group (0.61 ± 0.09) was lower than that in the R group (0.85 ± 0.04) on the 14th day (*p* = 0.0139). In addition, the FA value in the R group on the 14th day was markedly greater than that in the R group on the first (*p* < 0.001) and seventh (0.6 ± 0.1, *p* = 0.0173) days, with no significant difference compared with that in the S group (*p* > 0.05) on the 14th day. Moreover, there was a significant difference in the FA value between the R group and the RA (0.61 ± 0.05) group at 14 days (*p* = 0.0189). The difference in the FA value among the M, RA, and MA groups was not statistically significant (**Figure** **4A**).

Fiber tracking was performed using the Diffusion Toolkit (version 0.6.4.1) and TrackVis (version 0.6.1) software. The tract colors are defined by their FA values. DTT maps revealed that the fiber counts were obviously lower on day 1 postsurgery than those in the S group were (*p* < 0.05). The ratio of the fiber density in the ischemic region to that in the corresponding region increased over time in ischemic rats (**Figure** **4B**). Compared with those on the first day (0.51 ± 0.1 mm), the fiber counts in the R group were greater (0.87 ± 0.05 mm) than those on the first day (*p* < 0.001), and no significant difference was detected compared with those in the S group (*p* > 0.05). In addition, more fibers were observed in the R group than in the M (0.65 ± 0.05 mm) group on day 14 (*p* = 0.049). Moreover, there was a significant difference in fiber count between the R group and the RA (0.59 ± 0.14 mm) group at 14 days (*p* = 0.0051). These results suggested that rTMS could rescue MCAO‐induced decreases in fiber density in ischemic rats, but AMD3100 abrogated the beneficial effect of rTMS.

We also automatically analyzed the mean fiber length with software (**Figure** **4C**). The results revealed significant differences between the S group and the other groups on the first day, which were mostly decreased 7 days later. Compared with the S group, only the RA group presented significantly different mean fiber lengths on the 14th day (*p* = 0.0032). In addition, there was a significant difference in the mean fiber length between the R and RA groups (*p* = 0.0235), but no significant difference was observed among the M, R and MA groups after 14 days. Nonetheless, the values appeared to improve more in the rTMS group than in the other groups. Representative three‐dimensional fiber tracking maps for the three groups on days 1, 7, and 14 postocclusion are shown in **Figure** **4D**.

### 3.4 rTMS Alleviated Neurological Deficits After Focal Cerebral Ischemia in Rats

**FIGURE 5:**
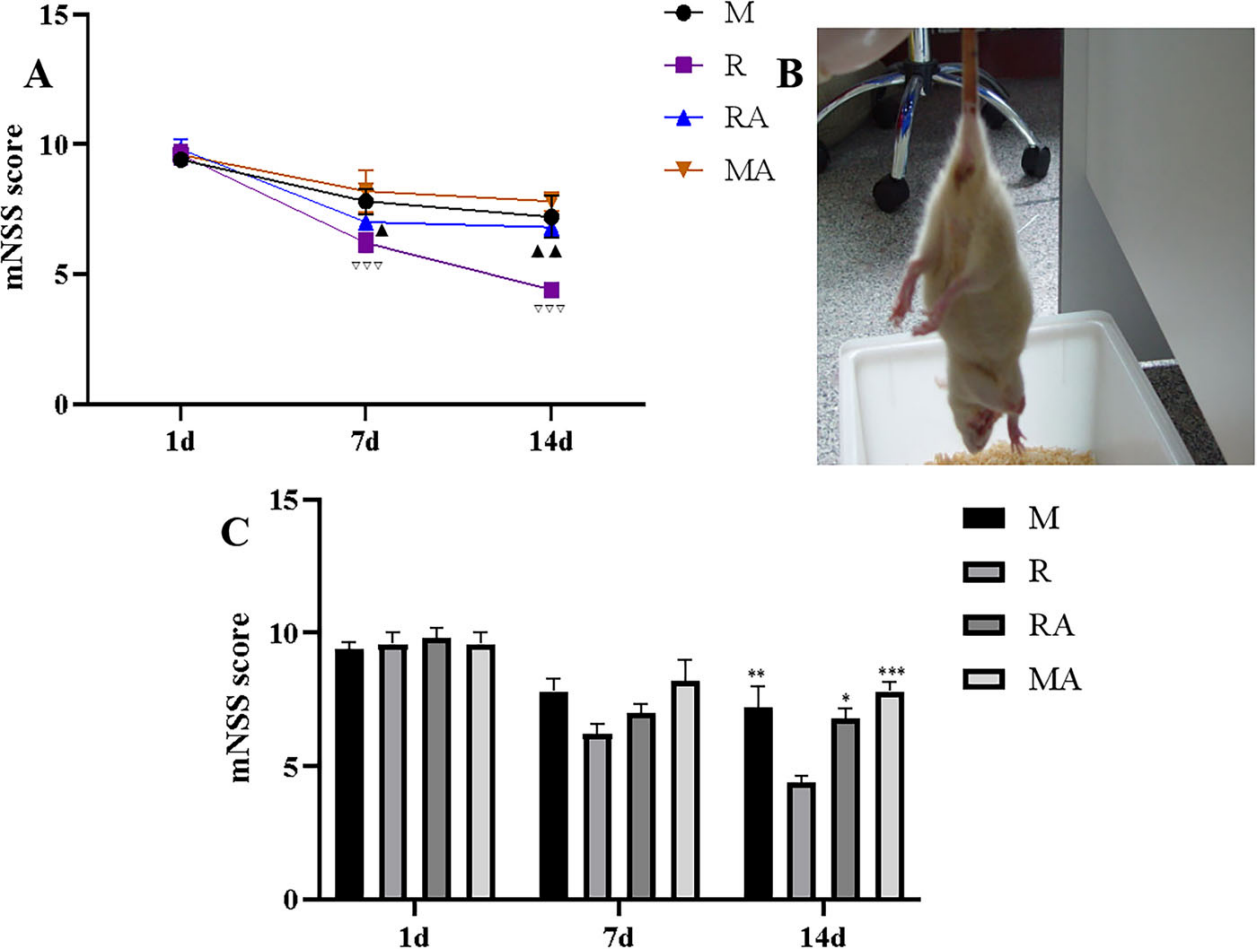
The effects of rTMS on the behavioral performance of rats at 1, 7, and 14 days after surgery. (A) The recovery of neurological function was evaluated by the mNSS. (B) Representative behavioral performance of the MCAO model rats. (C) Comparisons of the mNSSs among the four groups at different time points. ^▽▽▽^ *p* < 0.001 vs. the 1‐day R group; ^▲▲^ *p* < 0.01 and ^▲^ *p* < 0.05 vs. the 1‐day RA group; ^***^ *p* < 0.001, ^**^ *p* < 0.01 and ^*^ *p* < 0.05 vs. the R group.

To evaluate whether rTMS could alleviate neurological dysfunction in ischemic rats, we used the mNSS. The mNSS of the sham‐operated rats was 0. The animals subjected to MCAO exhibited severe neurological deficits 1 day after ischemia. No differences were observed among the ischemic rats first day (*p* > 0.05). Compared with that on the first day after surgery, the mNSS of the rTMS group was lower on both the seventh day and the 14th day (*p* < 0.01). Although the difference between the R (6.2 ± 0.8) and M (7.8 ± 1.1) groups on day 7 was not statistically significant (*p* = 0.4237), at the 14th day after surgery, the rats in the R (4.4 ± 0.5) group had progressive improvement compared with those in the M (7.2 ± 1.8) group (*p* = 0.0057). Moreover, there was a significant difference in the mNSS between the R group and the RA group (6.8 ± 0.84) at 14 days (*p* = 0.0319). In addition, as shown in Figure 5, the differences among the M, RA, and MA groups were not statistically significant on the seventh or 14th day (*p* < 0.05). These results suggested that rTMS effectively alleviated neurological deficits in MCAO rats, but AMD3100 abrogated the effect of rTMS at 14 days.

### 3.5 rTMS Improved Cognitive Function After Focal Cerebral Ischemia in Rats

**FIGURE 6:**
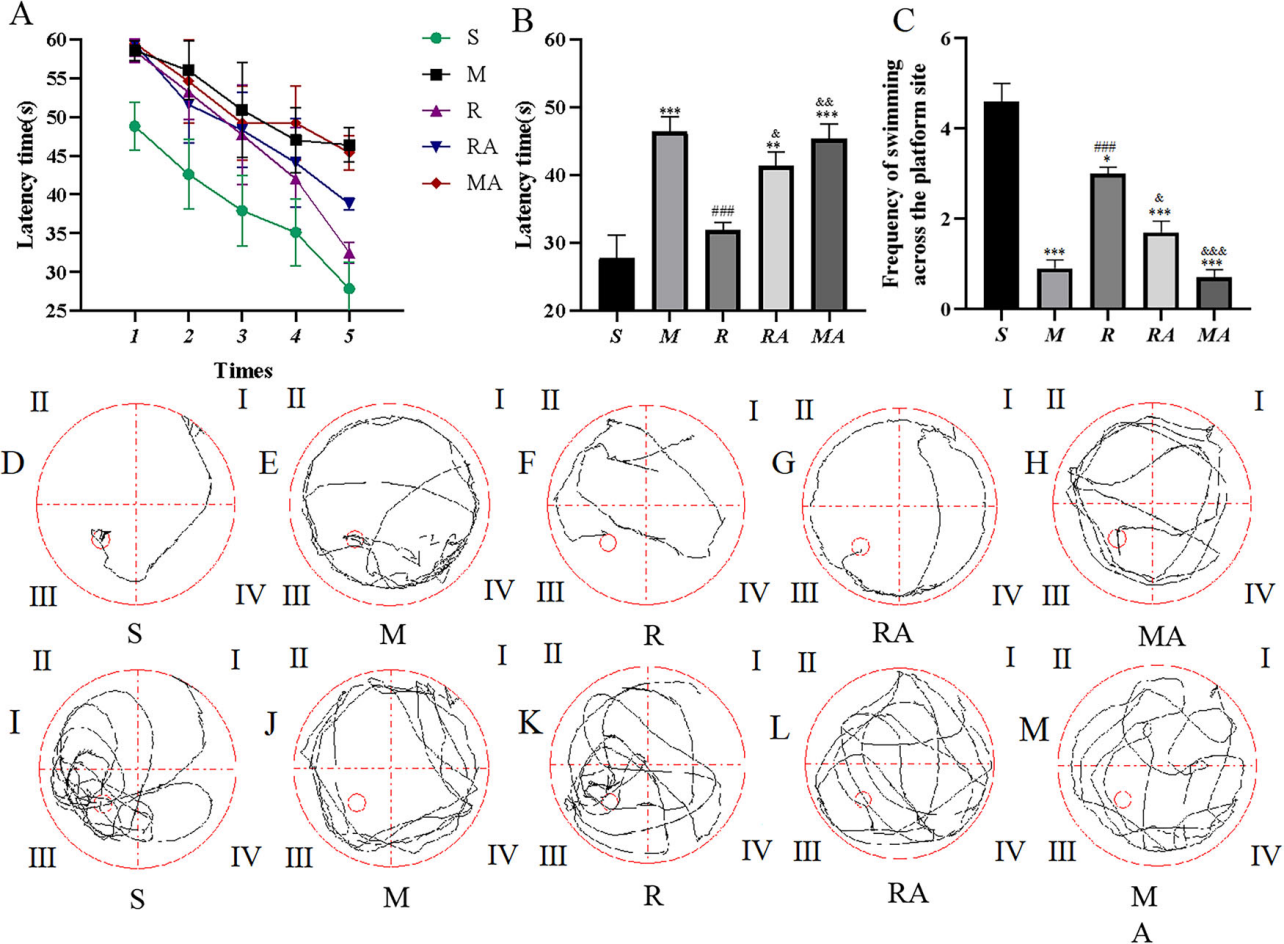
The effects of rTMS on cognitive impairment according to the MWM test. (A, B) Latency to find the submerged platform from the first day to the fifth day. (C) The number of platform site crossings in the spatial probe trial. (D–H) Representative trajectories of each group. (I–M) Representative trajectories showing the number of platform site crossings in the spatial probe trial. The gray lines indicate the swimming trajectories. ^***^ *p* < 0.001 vs. the S group; ^###^ *p* < 0.001 vs. the M group; ^&&^ *p* < 0.01 and ^&&&^ *p* < 0.001 vs. the R group.

Cognitive function was assessed by calculating the average latency to reach the platform and the number of platform crossings in the spatial probe trial. The changes in escape latency to find the submerged platform at the five time points in the MWM test were studied, and the escape latency was longer in the ischemic surgery groups than in the S group (*p* < 0.001) (**Figure** **6A**). In the last spatial probe trial, the escape latency was longer in the MCAO group (46.4 ± 6.95 s) than in the R group (31.9 ± 3.48 s) (*p* = 0.0006), as shown in **Figure** **6B**. In addition, compared with that of the rats in the RA (41.3 ± 6.9 s) group, the escape latency of the rats in the R group decreased (*p* = 0.04). In addition, no significant difference in escape latency was detected between the S group and R group. Typical swimming paths for each group are shown in **Figure** **6D**–**H**.

In addition, an analysis of the number of platform crossings during the 60 s observation period revealed that there were significant differences between the S group and the other groups (*p* < 0.05) (**Figure**  **6C**). The frequency of swimming across the platform was lower (*p* = 0.0396) in the R (3.1 ± 0.74) group than in the S (4.6 ± 1.84) group, but it was greater (*p* = 0.0008) in the R group than in the M (0.9 ± 0.88) group. Compared with those in the RA group (1.6 ± 1.07), the rats in the R group crossed the platform more times (*p* = 0.0396). Typical swimming paths for each group are shown in **Figure** 6I–M.

## 4 Discussion

Cognitive dysfunction is a common symptom observed in stroke survivors that significantly affects their quality of life, and early diagnosis of PSCI is necessary for assessing the need for rehabilitation. In the present study, we found that rTMS promoted structural remodeling after cerebral ischemia. We also demonstrated that 10 Hz rTMS attenuated cognitive deficits and motor impairments in MCAO model rats, and these effects were possibly associated with the activation of the SDF‐1α/CXCR4 axis. To our knowledge, this is the first study utilizing DTT in vivo to investigate the neuroprotective effects of rTMS against cerebral ischemia.

rTMS, a novel tool in the field of neurology with therapeutic benefits for recovery from stroke, is a promising approach for the treatment of PSCI. According to published clinical studies, the main mechanisms by which rTMS alleviates PSCI are the modulation of excitability in cortical areas and the promotion of plasticity in the cerebral cortex (Gong et al. 2023). In vivo studies have elucidated many mechanisms underlying the therapeutic effects of rTMS on PSCI. Wu et al. (2022) proposed that rTMS can ameliorate PSCI by modulating the levels of specific miRs. RNA sequencing and bioinformatics analysis suggested that the modulation of synaptic plasticity and chemical synaptic transmission in the hippocampus might be a possible mechanism underlying the effect of rTMS on PSCI (Hong et al. 2021). Hong et al. (2020) demonstrated that rTMS can modulate astrocyte polarization in cerebral ischemic models to promote recovery from PSCI. In addition, evidence that rTMS may alleviate PSCI by enhancing neurogenesis has also been reported (Guo et al. 2017). Most studies have suggested that rTMS exerts a positive effect on cognitive function.

Consistent with these positive results, we used a rat model of MCAO to confirm that rTMS ameliorates cognitive impairment. In addition, the results of our study confirmed that 10 Hz rTMS is safe and effective for alleviating PSCI. However, few studies have reported opposite results, claiming that rTMS has no evident therapeutic effect on PSCI patients. Therefore, further exploration of the mechanisms underlying the effect of rTMS is needed to better understand its effects on PSCI.

Studies have acknowledged that structural plasticity changes in the brain are closely related to the formation of new neural networks during recovery from stroke (Ueda et al. 2019). Many studies have confirmed that cognitive impairment is associated with the destruction and remodeling of white matter connections (Rashidi‐Ranjbar et al. 2020; Yang et al. 2022). White matter tracts are composed of a dense array of axons, which allow for rapid communication between various regions of the central and peripheral nervous systems. These tracts are sensitive to reduced local blood flow, which results in demyelination, swelling of myelin and destruction of axons. Ischemic stroke induces tissue infarction and the spread of damage to adjacent cells. White matter lesions are typical manifestations of ischemic damage, and severe lesions occur after cerebral ischemia (Guggisberg et al. 2019). According to previous studies, white matter lesions contribute to cognitive impairment after stroke, and patients with more severe white matter lesions are at greater risk of PSCI (He et al. 2022; Hong et al. 2022). Duru et al. (2016) reported a positive correlation between white matter changes and thalamic stroke. Yang et al. (2022) reported that white matter damage is significantly associated with PSCI in pMCAO model rats by DTI. Aleksonis et al. (2021) explored the relationship between cognitive function and white matter integrity by analyzing the correlation between FA and cognitive function and reported that the greater the FA is, the better the information processing speed, cognitive flexibility and delayed memory. Furthermore, a recent study revealed that repairing white matter lesions is a promising strategy for treating PSCI (Li et al. 2022). These previous results suggest that the repair of white matter structures adjacent to the stroke lesion may be vitally important to the response to rehabilitation interventions in PSCI patients.

As previously stated, rTMS is currently the method of choice for noninvasively inducing neural activity in the brain. The electric field it induces is strongest in the underlying white matter and may excite bands of myelinated axons in the juxtacortical white matter below the gyral crown. Auriat et al. (2015) first reported that rTMS treatment improved microstructures in motor‐related white matter brain regions that were correlated with behavioral recovery in stroke patients. Another study suggested that white matter, which modulates contralesional cortico‐cerebellar pathways and interhemispheric connections, may reflect contralesional compensation facilitated by excitatory rTMS (Li et al. 2018). Recently, one study indicated that the degree of motor function improvement is associated with the extent of white matter injury in motor function‐related areas and sensorimotor coordination‐related areas and that white matter nerve fiber structures are involved in functional improvement following rTMS (Ueda et al. 2021). Some studies have also shown that rTMS may mitigate white matter damage by modulating neuroinflammatory responses, promoting remyelination, and facilitating axonal repair (Sheng et al. 2023). Hence, white matter structural improvements may underlie the beneficial effect of rTMS after ischemic stroke.

DTI is a noninvasive approach for visualizing and analyzing white matter integrity. DTT, also known as fiber tractography, is a noninvasive imaging method that shows the travel and spatial distribution of white matter fiber bundles three‐dimensionally in vivo and can reflect the structure of the whole brain and the connectivity of different brain regions (Zhang et al. 2019). Therefore, DTI and DTT are effective techniques for visualizing structural changes after rTMS and for elucidating the therapeutic mechanism of rTMS in stroke recovery. In fact, DTI has been widely used to analyze the correlation between white matter changes and functional responses in stroke patients. DTI revealed that, compared with acupuncture alone, acupuncture combined with rTMS significantly promotes white matter tract restoration and improves the ability to perform activities of daily living (ADL) in stroke patients (Zhao et al. 2018). Another preliminary DTI study revealed that rTMS‐treated stroke patients exhibited improvements in motor function and alleviation of motor‐related white matter lesions (Guo et al. 2016).

However, much less is known about the role of white matter lesions in the ability of rTMS to promote recovery from PSCI. Only one recent study indicated that rTMS improves cognitive function and attenuates white matter lesions via Luxol fast blue (LFB) staining in rats after cerebral ischemia (Chen et al. 2023). Another recent study by the same team investigated the positive effects of rTMS on white matter integrity by DTI‐based tract‐based spatial statistics (Chen et al. 2025). In our research, the FA findings are consistent with their results. Innovatively, our study also revealed white matter fibers through DTT images. The white matter fibers in the ischemic hemisphere were sparser than those in the contralateral hemisphere, with abnormal morphology and partial curling, and some white matter fibers were missing in all ischemic rats on the first, seventh and 14th days after stroke. The statistical results revealed that the mean tract length and density were significantly decreased after MCAO and were increased by rTMS treatment on the 14th day. The results showed that rTMS could alleviate nerve fiber injury and protect the integrity of white matter nerve fiber bundles after ischemic stroke. Structural improvements in white matter induced by rTMS represent potential means for the recovery of cognitive functions following ischemic injury.

In addition, dynamic changes in the expression of SDF‐1α/CXCR4 axis components in ischemic tissues following rTMS intervention may help explain the therapeutic effect of this approach. SDF‐1α is constitutively expressed in the normal nervous system, and its expression may be significantly induced by ischemia, a hypothesis supported by our results. Consistent with our findings, the expression of SDF‐1α within white matter structures was also found to increase following ischemic injury (Miller et al. 2005). Furthermore, cognitive function recovery may result from elevated levels of SDF‐1α and CXCR4 in ischemic rats (Zangenberg et al. 2020). Additionally, our previous study revealed that 10 Hz rTMS can significantly increase the protein expression levels of SDF‐1α and CXCR4 in the striatum of MCAO model rats, which contributes to the restoration of motor function (Deng et al. 2021). Accumulating evidence indicates that SDF‐1α is involved in axonal pathfinding, outgrowth, and branching, whereas axonal sprouting occurs from the intact cortex to the peri‐injured cortex and striatum (Beigi Boroujeni et al. 2020). Studies have indicated that SDF‐1α/CXCR4 signaling is a key mechanism by which white matter tracts are utilized by neural progenitor cells for efficient movement (Chen et al. 2015). A correlation between the recovery of neurological function and increased SDF‐1α and CXCR4 expression in rats with white matter damage was reported by Li et al. (2015). In addition, Yin et al. (2020) reported that SDF‐1α gene therapy can protect myelin sheath integrity in the perifocal region, whereas coadministration of AMD3100 abolishes the beneficial effect of SDF‐1α on myelin sheath integrity. White matter lesions are thought to represent regions of axon loss, demyelination and myelin swelling. Hence, it is theoretically possible that rTMS regulates structural plasticity and promotes behavioral recovery by increasing the expression of SDF‐1α/CXCR4 axis components in ischemic brain tissues after stroke. In the present study, we observed that the protein and mRNA levels of SDF‐1α and CXCR4 were greater in ipsilateral brain tissues after rTMS. rTMS reduced cerebral infarct severity, ameliorated white matter injury and promoted behavioral recovery after stroke in addition to increasing SDF‐1α and CXCR4 expression. FA values and fiber density markedly improved after rTMS treatment. However, blocking the SDF‐1α/CXCR4 signaling pathway with AMD3100 abrogated the effects of rTMS on behavioral recovery and white matter injury. The difference in the mean fiber length among the M, R and MA groups was not significant on either day 7 or day 14 postsurgery. This might have been due to the variation in the minimum and maximum fiber lengths in the corresponding ischemic regions. The precise expression of SDF‐1α and CXCR4 in ischemic stroke and the specific mechanism involved in remyelination require further exploration.

The major aim of the present study was to investigate the mechanisms underlying the neuroprotective efficacy of rTMS on PSCI. Our results provide several findings demonstrating that rTMS relieves structural impairments in ischemic brain tissues, possibly through the SDF‐1α/CXCR4 signaling pathway. First, the results of T2WI revealed that applying 10 Hz rTMS to MCAO model rats ameliorated infarct severity. Second, the DTI results revealed that applying 10 Hz rTMS to MCAO model rats attenuated white matter lesions. Moreover, according to the mNSS scale and MWM test, the application of 10 Hz rTMS to MCAO model rats improved behavior. These positive effects were abolished by the CXCR4 inhibitor AMD3100. All of our data support the notion that rTMS is an efficient treatment for PSCI and that the effects of rTMS on these symptoms might involve increasing SDF‐1α/CXCR4 levels in ischemic brain tissues.

## 5 Conclusion

Taken together, these findings indicate that the ability of rTMS to promote the recovery of neurological function after focal cerebral ischemia is associated with the activation of the SDF‐1α/CXCR4 axis. This could mediate, at least in part, the beneficial effects of rTMS on structural plasticity and PSCI in the brain after transient focal cerebral ischemia. The findings are novel in that no one has previously described how these white matter structures change on DTT in response to rTMS in vivo. Our study provides new insights into the effects of rTMS on neurological deficits after ischemic stroke. However, 3T MRI in DTI, including susceptibility to geometric distortions and relatively lower spatial resolution compared to high‐field systems, 5T or better technology would help us to more clearly in fiber tracking results. Besides, more evidence, such as from electron microscopy, LFB staining, immunostaining of OPCs and OLs, or some markers of white matter repair, are needed to confirm these preliminary findings. Meanwhile, further elaborate on the potential mechanism of action of SDF‐1α/CXCR4 in white matter remodeling, as well as the specific contribution of activation of this signaling pathway to neuroprotection and improvement of cognitive function, need to be explored in the future experiments. Although the results of our study are positive, further investigations with larger sample sizes are necessary to obtain more reliable results. Deeper clinical research could confirm the effects of rTMS on PSCI, white matter integrity and the SDF‐1α/CXCR4 axis after stroke.

## Author Contributions

FG designed the research, guided the experiments, and wrote the manuscript. XXH conducted the animal models, the rTMS treatment, behavior tests, and analyzed the data. QL and CL constructed the animal models and conducted WB and PCR testing. XYT and HYS completed the MRI scan. All authors have read and approved the final manuscript.

## Funding

This study was supported by China's National Natural Science Foundation (Nos: 81702231 and 51907077).

## Conflicts of Interest

The experimental designs and all procedures were in accordance with the National Institutes of Health Guide for the Care and Use of Laboratory Animals. All animal experiments were approved by the ethics committee of the Tongji Medical College (Permit Number: 298). Utmost efforts were made to minimize the number of animals used and their sufferings. We confirm that the study is in accordance with ARRIVE guidelines.

## Data Availability

The datasets generated during and/or analyzed during the current study are available from the corresponding author on reasonable request.
